# Split application of polymer-coated urea combined with common urea improved nitrogen efficiency without sacrificing wheat yield and benefits while saving 20% nitrogen input

**DOI:** 10.3389/fpls.2024.1321900

**Published:** 2024-02-05

**Authors:** Quan Ma, Rongrong Tao, Wenxin Jia, Min Zhu, Jinfeng Ding, Chunyan Li, Wenshan Guo, Guisheng Zhou, Xinkai Zhu

**Affiliations:** ^1^ Jiangsu Key Laboratory of Crop Genetics and Physiology, Agricultural College of Yangzhou University, Yangzhou, China; ^2^ Co-Innovation Center for Modern Production Technology of Grain Crops, Yangzhou University, Yangzhou, China; ^3^ Joint International Research Laboratory of Agriculture and Agri-Product Safety, The Ministry of Education of China, Yangzhou University, Yangzhou, China

**Keywords:** polymer-coated urea, wheat, yield, nitrogen use efficiency, benefit, nitrogen-saving

## Abstract

Controlled-release nitrogen fertilizer (CRNF) has been expected to save labor input, reduce environmental pollution, and increase yield in crop production. However, the economic feasibility is still controversial due to its high cost. To clarify the suitable application strategy of CRNF in promoting the yield, nitrogen use efficiency and income on wheat grown in paddy soil, four equal N patterns were designed in 2017−2021 with polymer-coated urea (PCU) and common urea as material, including PCU applied once pre-sowing (M1), PCU applied 60% at pre-sowing and 40% at re-greening (M2), 30% PCU and 30% urea applied at pre-sowing, 20% PCU and 20% urea applied at re-greening (M3), and urea applied at four stage (CK, Basal:tillering:jointing:booting=50%:10%:20%:20%). In addition, M4−M6, which reduced N by 10%, 20% and 30% respectively based on M3, were designed in 2019−2021 to explore their potential for N-saving and efficiency-improving. The results showed that, compared with CK, M1 did not significantly reduce yield, but decreased the average N recovery efficiency (NRE) and benefits by 1.63% and 357.71 CNY ha^−1^ in the four years, respectively. M2 and M3 promoted tiller-earing, delayed the decrease of leaf area index (LAI) at milk-ripening stage, and increased dry matter accumulation post-anthesis, thereby jointly increasing spike number and grain weight of wheat, which significantly increased yield and NRE compared with CK in 2017−2021. Due to the savings in N fertilizer costs, M3 achieved the highest economic benefits. With the 20% N reduction, M5 increased NRE by 16.95% on average while decreasing yield and net benefit by only 6.39% and 7.40% respectively, compared with M3. Although NRE could continue to increase, but the yield and benefits rapidly decreased after N reduction exceeds 20%. These results demonstrate that twice-split application of PCU combined with urea is conducive to achieving a joint increase in yield, NRE, and benefits. More importantly, it can also significantly improve the NRE without losing yield and benefits while saving 20% N input.

## 1 Introduction

Nitrogen (N) as the most essential nutrient for crop growth, often requires a large amount of additional artificial supplement in wheat (*Triticum aestivum* L.) cultivation to achieve high yield (Cai et al., 2002; Zheng et al., 2020). However, the application of traditional N fertilizer can easily lead to a rapid increase in soil N concentration during a short period, increasing the risk of N loss through denitrification, ammonia volatilization, and leaching (Hodge et al., 2000; Selbie et al., 2015). Moreover, excessive investment and improper application methods of N fertilizer by farmers in production further exacerbate N loss, which not only reduces N use efficiency (NUE), but also causes serious resource waste and environmental pollution (Jiang et al., 2010; Yang et al., 2015). To improve NUE and reduce the risk of reactive N loss, the traditional fertilization strategy in wheat production is to apply N fertilizer in 3−4 times to alleviate the accumulation of nitrate in the soil and promote the absorption and utilization of N by plants (Hao et al., 2023). However, the agricultural labor force is increasingly scarce, while the multiple application of quick-acting N fertilizer requires excessive labor input, which leads to a decrease in benefit return, and thus reduces the farmers’ willingness to grow wheat (Zheng et al., 2016a; Fan et al., 2022). It is urgent to reduce dependence on N fertilizer inputs to promote the sustainability of agricultural production. As a result, exploring reasonable fertilization strategies has become an important research direction in wheat production to ensure the maximum NUE and wheat yield, while improving economic benefits and alleviating environmental pollution without increasing N fertilizer dosage and fertilization frequency.

In recent years, controlled-release nitrogen fertilizer (CRNF) has attracted much attention in the agricultural field due to its efficient and environmental friendly characteristics (Trenkel, 2010). By wrapping different coatings on the surface of urea particles, CRNF can slowly and continuously release nutrients after being applied to the soil, which can meet the nutrient demand of crops while controlling the nitrate concentration in the soil (Shoji et al., 2001; Liu et al., 2019a). Many studies have confirmed that the application of CRNF could not only reduce N loss through various pathways, but also promote N absorption and utilization by crops, thus increasing NUE and reducing environmental burden compared with urea (Ji et al., 2012; Gao et al., 2018; Cui et al., 2022). There is no doubt that CRNF has the potential to save labor input and reduce environmental burden in crop planting. Nevertheless, it is still a great challenge to persuade farmers to apply CRNF in food crop production due to the high cost of CRNF (Davidson and Gu, 2012; Mikula et al., 2020). Furthermore, winter wheat usually has a long growth period (usually exceeding 200 d), and the temperature and precipitation fluctuate greatly during the wheat growth period, which may affect the effectiveness of CRNF. Therefore, it is still controversial whether one-time application of CRNF can meet the N demand of wheat during the whole growth period (Zhao et al., 2020; Ma et al., 2021).

Some reports have shown that one-time application of CRNF was beneficial for wheat yield formation compared to the multiple application of urea (Zheng et al., 2016a; Fan et al., 2021; Shen et al., 2022). However, Farmaha and Sims (2013a) argued that due to the slow release of N in the early stage, one-time application of CRNF pre-sowing failed to provide sufficient nitrogen source for wheat. Several studies have also pointed out that there are two N demand peaks in winter wheat during the seedling stage and the jointing to booting stage (Liu et al., 2019b; Cui et al., 2022). Due to the limited of nutrient release cycle, N release from one-time application of CRNF is difficult to cover the entire wheat growth period. As a result, although one-time application of CRNF can satisfy N supply for tiller differentiation at the seedling stage, it easily leads to N deficiency in the later stage of wheat, which limits the matter accumulation and grain filling post-anthesis (Ma et al., 2022). The study on wheat grown in paddy soil by Ji et al. (2012) also confirmed that one-time application of CRNF reduced the volatilization of NH_3_ and the emissions of N_2_O in wheat fields compared to common urea, but did not significantly improve the wheat yield and NUE. To overcome the high cost of CRNF, some scholars have proposed the mixed application of CRNF and urea, which is believed to be conducive to alleviating the decrease in soil pH, increasing soil exchangeable cations (Zheng et al., 2017), regulating soil microbial activity (Li et al., 2021a), alleviating the side effects of short-term N fixation caused by straw returning to the field (Alijani et al., 2012), and improving the wheat yield and NUE (Zheng et al., 2016b).

In addition, many studies have put forward that the reduction application of CRNF combined with urea has the potential to reduce active N loss and promote N balance during the wheat growing season (Ji et al., 2012; Xue et al., 2014; Gao et al., 2018). However, the appropriate N reduction range for CRNF without sacrificing yield in wheat production has not yet been clarified. Our previous study has confirmed that twice-split application of CRNF was conducive to promoting the synchronization of fertilizer N supply with wheat N demand, which could improve N absorption and utilization by plants, thereby increasing NUE and yield (Ma et al., 2021). Nevertheless, we have not clarified the application effect, economic feasibility and N-saving potential of twice-split application of CRNF combined with urea in wheat production. Therefore, several application patterns were designed with polymer-coated urea (PCU) as test material based on the actual needs of labor saving, cost reduction, yield increasing, and fertilizer saving in production, and their effects on wheat agronomic traits, N absorption and utilization, yield and its components, NUE, and economic benefits were determined. The purposes of this study were to (1) investigate the regulatory effects of different application modes of PCU on wheat growth, and the potential of PCU for N reducing and efficiency enhancement; (2) clarify the optimal application strategy of PCU and its mechanisms for promoting the wheat yield and increasing NUE, and explore its economic feasibility. This study offers new insights into for promoting high-yielding and high-efficiency cultivation of wheat, and provides a theoretical basis for reducing agricultural N input and energy waste, thereby ensuring world food security.

## 2 Materials and methods

### 2.1 Experimental site and materials

**Figure 1:**
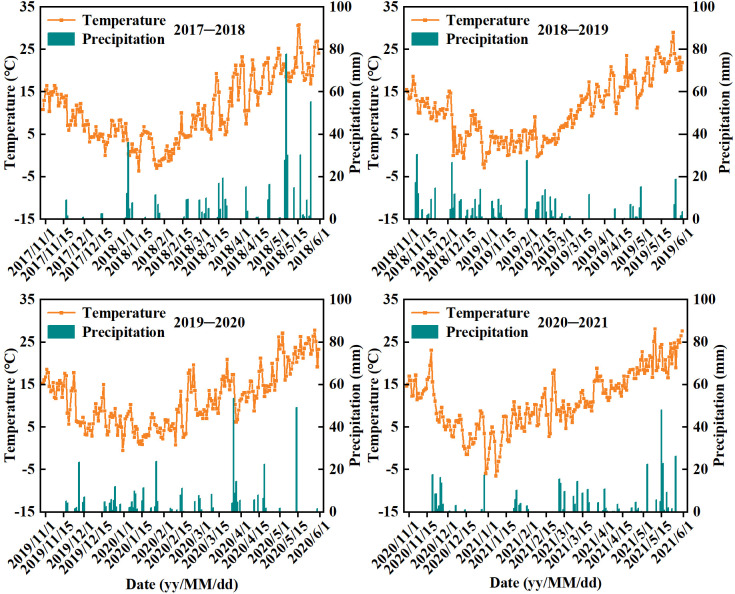
Monthly mean temperature and total precipitation amount during the wheat growing season at the test site in 2017−2021.

The field experiment was conducted in four wheat growing seasons in 2017−2021 at the Agricultural Experiment Station of Jiangsu Key Laboratory of Crop Genetics and Physiology, Yangzhou University (32°39′N, 119°42′E), which has a humid subtropical climate (**Figure 1**). The test site is a typical summer rice (*Oryza sativa* L.) -winter wheat rotation system with sandy loam soil. The basic soil properties of the cultivated layer (0−20 cm) before sowing in the test site were shown in **Table 1**. The wheat cultivar selected in this study was Yangmai 23, a widely grown variety locally, which was bred by the Institute of Agricultural Sciences of Lixiahe, Jiangsu Province. The CRNF chosen in this study was PCU (45% N), with a nutrient controlled-release period of 90−120 d, which was produced by Hanfeng Slow-Release Fertilizer (Jiangsu) Co., Ltd., China. In our previous study (Ma et al., 2021), the release rate curve of polymer-coated urea has been determined in wheat field, which exhibited a sigmoidal release pattern. The N release rate of PCU increased continuously at first and then decreased gradually. The conventional fertilizers used in this study included urea (46.3% N), superphosphate (12% P_2_O_5_) and potassium chloride (60% K_2_O).

**Table 1:** Primary properties of topsoil (0−20 cm) pre-sowing at the test field.

Year	Organic matter (g kg^−1^)	Total N (g kg^−1^)	Available N (mg kg^−1^)	Available P (mg kg^−1^)	Available K (mg kg^−1^)	pH
2017−2018	14.14	1.06	65.52	42.25	112.76	7.16
2018−2019	14.20	1.02	74.20	47.60	102.00	7.27
2019−2020	20.65	1.08	81.60	65.28	138.21	7.27
2020−2021	16.22	0.94	73.55	67.72	92.02	6.82

### 2.2 Experimental design and field management

A single-factor randomized block was designed in this test with three replications. In 2017−2019, three patterns (M1−M3) with equal N rate (225 kg ha^−1^ N) were designed using PCU and urea, and urea applied fourth (225 kg ha^−1^ N) was taken as the control (CK). A blank control with the same phosphorus and potassium fertilizers but without N application was also set up to calculate N efficiency. Based on the experimental results in 2017−2019, the optimal application pattern for yield, NUE and benefits was selected (M3). And then three additional N reduction treatments (M4−M6) were added according to M3 in 2019−2021 (**Table 2**). The phosphorus (P_2_O_5_) and potassium (K_2_O) fertilizers were applied once before sowing at the rate of 112.5 kg ha^−1^.

**Table 2:** Fertilization patterns design scheme.

Treatment	N rate	N management
CK	225.0 kg N ha^−1^	100% N-U, Basal:tillering:jointing:booting=50%:10%:20%:20%
M1	225.0 kg N ha^−1^	100% N-PCU applied once before sowing
M2	225.0 kg N ha^−1^	60% N-PCU applied before sowing, 40% N-PCU topdressing at re-greening stage
M3	225.0 kg N ha^−1^	30%N-PCU combined with 30%N-U applied before sowing, 20%N-PCU combined with 20%N-U topdressing at re-greening stage
M4	202.5 kg N ha^−1^	M3 reducing N by 10%
M5	180.0 kg N ha^−1^	M3 reducing N by 20%
M6	157.5 kg N ha^−1^	M3 reducing N by 30%

Each block was designed with an area of 16.2 m^2^ (2.7 m × 6 m). On November 3, 2017, November 1, 2018, October 30, 2019, and October 31, 2020, a plot seeder was used to sow seeds (135 kg ha^−1^) with a row spacing of 27 cm. At the three-leaf stage, the density of seedlings in each plot was uniformly adjusted to 225×10^4^ plant ha^−1^ after removing excess seedlings manually. Other field management followed conventional high-yield cultivation measures.

### 2.3 Samplings and measurements

#### 2.3.1 Yield and yield components

At the milk-ripening stage (Zadoks growth stage, GS75), 50 ears were consecutively taken from each plot and the number of grain per ear was counted. In each plot, 1.08 m^2^ (four rows with a length of 1 m) was randomly designated at the maturity stage (GS92), and then harvested and threshed after counting the spike number. The weight and moisture content of grains were calculated after natural drying. A total of 1000 grains were counted randomly to determine the 1000-grain weight. The grain yield and 1000-grain weight were converted into the standard weight at a moisture content of 13%, respectively (Ding et al., 2023).

#### 2.3.2 Dynamics of stem and tiller number, dry matter accumulation, and leaf area index

A total of 20 plants were sampled from each plot at the key growth stages of wheat: over-wintering (GS16), jointing (GS32), booting (GS45), anthesis (GS60), and maturity (GS92). The stem and tiller number was counted, and the leaf area was measured to calculate leaf area index (LAI). After being divided into different organs (stem and sheath, leaf, ear axis and glume, grain), the samples were first placed in an oven at 105°C for 1 h to deactivate enzymes, and then dried at 80°C to constant weight to determine dry matter accumulation (DMA).

#### 2.3.3 Total grain number

The total grain number was calculated according to the following formula (Equation 1) (Ukozehasi et al., 2022):


(1) $$\text{Total grain number }\left({\times 10}^{4}{\text{ ha}}^{-1}\right)=\text{Spikes per unit area}\times \text{Grains per spike}$$

#### 2.3.4 Nitrogen uptake and nitrogen use efficiency

The dried plant samples were crushed and passed through a 2-mm sieve, and N concentration in each organ of the plant was determined by the Kjeldahl method (Douglas et al., 1980). The plant N accumulation at maturity was the sum of the product of N concentration in each aboveground organ and dry matter accumulation. N recovery efficiency (NRE) and N agronomic efficiency (NAE) were calculated as follows (Equations 2, 3) (Thilakarathna et al., 2020):


(2) $$\text{NRE }\left(\%\right)=\frac{\text{N uptake in N treatment}-\text{N uptake in N}0\text{ treatment}}{\text{N application rate}}\times 100\%$$

(3) $$\text{NAE }\left({\text{kg kg}}^{-1}\right)=\frac{\text{Grain dry matter in N treatment}-\text{Grain dry matter in N}0\text{ treatment}}{\text{N application rate}}$$

Where N uptake was N accumulation of wheat at maturity; N treatment was the treatment with N application in the experiment; Grain dry matter was grain dry weight at maturity; N_0_ treatment was the blank control with the same phosphorus and potassium fertilizers but without N application.

### 2.4 Data processing and statistical analysis

To evaluate the economic benefits of different treatments, the wheat output and net benefit were calculated by the following formula (Equations 4, 5) (Ma et al., 2022):


(4) $$\text{Total output }\left({\text{CNY ha}}^{-1}\right)=\text{grain yield}\times \text{unit price of wheat}$$

(5) $$\text{Net benefit }\left({\text{CNY ha}}^{-1}\right)=\text{Total output}-{\text{C}}_{\text{NF}}-{\text{C}}_{\text{TL}}-{\text{C}}_{\text{OT}}$$

Where C_NF_ is the N fertilizer cost, C_TL_ is the topdressing labor cost, and C_OT_ is the sum of other costs.

The price of wheat was calculated based on the average price in Jiangsu Province in the current year, which was 2330.3 CNY t^−1^, 2276.9 CNY t^−1^, 2424.3 CNY t^−1^ and 2291.3 CNY t^−1^ in the four years respectively. The price of PCU was 3500 CNY t^−1^ in 2017−2019 and 4200 CNY t^−1^ in 2019−2021, while common urea was 2100 CNY t^−1^ in 2017−2019 and 2700 CNY t^−1^ in 2019−2021. Other costs mainly included phosphorus and potassium fertilizers (630 CNY ha^−1^), seeds (546 CNY ha^−1^), pesticides (420 CNY ha^−1^), machinery (1300 CNY ha^−1^), and labor (2400 CNY ha^−1^, excluding topdressing), totaling 5926 CNY ha^−1^. The cost of artificial topdressing was 150 CNY ha^−1^ per time, which was in line with the marketing price in the region (Sheng et al., 2022).

Excel 2010 was used for data processing, Origin 95 (Origin Lab, USA) was used for drawing, and DPS 7.05 (Zhejiang University, Hangzhou) was adopted for one-way analysis of variance (ANOVA). Duncan’s multiple range test (*p* < 0.05) was selected for significance test by means comparison.

## 3 Results

### 3.1 Response of wheat yield and yield components to different nitrogen treatments

With the equal N application rate, the yield in different patterns was in order of M3 > M2 > M1 (**Table 3**). The yield in M3 and M2 was significantly higher than that in CK, with an average increase of 12.92% (10.76%−17.44%) and 10.83% (8.83%−15.11%) in the four years, respectively. In terms of yield components, M2 and M3 achieved the highest spike number and 1000-grain weight, which could explain the increase in grain yield. M1 had the lowest spike number and 1000-grain weight, which was significantly lower than M2 and M3 in 2017−2020.

**Table 3:** Grain yield and yield components of wheat in different N treatments in 2017–2021.

Year	Treatment	Spike number (×10^4^ ha^−1^)	Grains per spike	1000-grain weight (g)	Yield (kg ha^−1^)	Yield increase *vs*. CK (%)
2017−2018	CK	521.30 ± 9.40b	44.19 ± 0.43a	33.83 ± 0.03b	7174.70 ± 118.84b	−
	M1	514.20 ± 9.27b	44.85 ± 0.28a	33.95 ± 0.16b	7367.54 ± 99.15b	2.69
	M2	545.06 ± 7.71a	43.07 ± 0.49a	36.43 ± 0.02a	8258.81 ± 525.17a	15.11
	M3	542.59 ± 6.42a	44.55 ± 1.39a	36.28 ± 0.03a	8426.06 ± 353.18a	17.44
2018−2019	CK	465.00 ± 6.57b	43.24 ± 1.41a	45.51 ± 0.17b	8595.31 ± 269.79b	−
	M1	443.00 ± 10.39c	44.46 ± 0.59a	45.38 ± 0.14b	8526.12 ± 182.41b	−0.80
	M2	490.33 ± 5.25a	42.14 ± 0.20a	48.04 ± 0.12a	9456.51 ± 222.93a	10.02
	M3	486.67 ± 14.13a	44.56 ± 0.45a	47.95 ± 0.30a	9603.36 ± 262.52a	11.73
2019−2020	CK	419.14 ± 7.87c	45.08 ± 1.02ab	49.15 ± 0.04c	8918.83 ± 422.09c	−
	M1	418.83 ± 16.83c	47.50 ± 0.20a	46.45 ± 0.25f	8842.86 ± 234.87c	−0.85
	M2	457.10 ± 17.13ab	44.80 ± 1.41ab	51.11 ± 0.18a	9753.75 ± 251.66ab	9.36
	M3	470.06 ± 13.30a	45.46 ± 1.61ab	49.65 ± 0.13b	9966.90 ± 422.04a	11.75
	M4	466.05 ± 10.65ab	45.40 ± 0.06ab	48.52 ± 0.02d	9712.25 ± 270.09ab	8.90
	M5	464.20 ± 8.60ab	44.06 ± 1.78b	47.82 ± 0.13e	9442.50 ± 252.09b	5.87
	M6	433.64 ± 31.97bc	42.94 ± 1.16b	48.2 ± 0.24de	8633.64 ± 204.12c	−3.20
2020−2021	CK	409.88 ± 16.70ab	44.18 ± 0.54ab	46.35 ± 0.08d	7847.80 ± 250.44c	−
	M1	398.98 ± 13.33b	45.02 ± 0.42a	44.33 ± 0.11f	7715.24 ± 332.32cd	−1.69
	M2	427.38 ± 8.85ab	42.90 ± 1.39bc	48.99 ± 0.33a	8540.67 ± 146.62ab	8.83
	M3	432.53 ± 9.08a	43.60 ± 0.23abc	48.46 ± 0.05b	8691.85 ± 319.12a	10.76
	M4	423.70 ± 22.27ab	43.26 ± 0.08bc	47.17 ± 0.14c	8426.49 ± 283.34ab	7.37
	M5	414.54 ± a19.23b	42.98 ± 0.14bc	46.14 ± 0.14d	8037.51 ± 358.96bc	2.42
	M6	400.49 ± 17.65b	41.95 ± 0.35c	45.09 ± 0.14e	7233.15 ± 356.53d	−7.83

Means within each column and year followed by different lowercase letters were significant at *p*< 0.05.

The grain yield, spike number, grain number per spike and 1000-grain weight all showed a decrease trend with the reduction of N application rate (**Table 3**). Compared with that in M3, the yield in M4, M5 and M6 decreased by 2.55%, 5.26% and 13.38% respectively in 2019−2020, as well as 3.05%, 7.53% and 16.78% respectively in 2020−2021. Compared with CK, M4 and M5 increased yield by 8.13% and 4.14% respectively in the two years. While M6 decreased the yield by 5.51% on average in the two years compared with CK.

### 3.2 Response of wheat agronomic traits to different nitrogen treatments

#### 3.2.1 Dynamics of stem and tiller number

With the equal N application rate, there were significant differences in the stem and tiller number in different treatments (**Table 4**). M1 achieved the highest stem and tiller number in both over-wintering and jointing stages, but decreased rapidly after jointing, resulting in the lowest spike number at the maturity stage. Compared with CK, M1 decreased the spike number at the maturity stage by 1.64% on average in 2017−2021. The stem and tiller number in M3 tended to be lower than that in CK at the over-wintering stage, but was obviously higher at the maturity stage, with an average increase of 6.81% in the four years. With N reduction by 10%−20%, the stem and tiller number in M4−M5 showed a decreasing trend compared with M3, but all showed no significant difference at different stages. The stem and tiller number in M6 had no significant difference at the over-wintering stage, but was significantly lower than that in M3 from jointing to maturity.

**Table 4:** Dynamics of stem and tiller number of wheat in different N treatments in 2017–2021.

Year	Treatment	Over- wintering (×10^4^ ha^−1^)	Jointing (×10^4^ ha^−1^)	Booting (×10^4^ ha^−1^)	Anthesis (×10^4^ ha^−1^)	Maturity (×10^4^ ha^−1^)
2017−2018	CK	698.8 ± 47.8a	1177.8 ± 45.5a	882.7 ± 19.0a	681.5 ± 16.1a	521.3 ± 9.4b
	M1	745.7 ± 15.4a	1180.2 ± 4.3a	758.0 ± 36.4c	622.2 ± 7.4c	514.2 ± 9.3b
	M2	692.6 ± 29.4a	1125.9 ± 38.5a	802.5 ± 21.4bc	643.2 ± 5.7bc	545.1 ± 7.7a
	M3	719.8 ± 37.3a	1127.2 ± 37.5a	842.0 ± 20.4ab	654.3 ± 26.0ab	542.6 ± 6.4a
2018−2019	CK	761.7 ± 30.8ab	835.8 ± 26.3a	708.6 ± 10.7a	549.4 ± 22.3ab	465.0 ± 6.6b
	M1	780.2 ± 36.0a	898.8 ± 35.6a	676.5 ± 21.7a	516.0 ± 2.1b	443.0 ± 10.4c
	M2	700.0 ± 30.3b	838.3 ± 15.0a	685.2 ± 3.7a	569.1 ± 24.1a	490.3 ± 5.2a
	M3	727.2 ± 17.5ab	864.2 ± 38.5a	712.3 ± 21.7a	560.5 ± 11.9a	486.7 ± 14.1a
2019−2020	CK	696.5 ± 18.1ab	−	−	494.8 ± 15.0c	419.1 ± 7.9c
	M1	717.7 ± 16.9a	−	−	480.9 ± 16.0c	418.8 ± 16.8c
	M2	677.0 ± 21.7ab	−	−	531.6 ± 11.6b	457.1 ± 17.1ab
	M3	687.4 ± 22.4ab	−	−	561.4 ± 7.5a	470.1 ± 13.3a
	M4	685.0 ± 21.8ab	−	−	545.9 ± 18.3ab	466.0 ± 10.7ab
	M5	675.3 ± 11.2ab	−	−	531.8 ± 17.8b	464.2 ± 8.6ab
	M6	656.8 ± 14.8b	−	−	496.5 ± 16.8c	433.6 ± 32.0bc
2020−2021	CK	623.1 ± 19.6ab	824.1 ± 35.1bc	604.2 ± 21.1abc	473.8 ± 14.6bcd	409.9 ± 16.7ab
	M1	656.5 ± 36.7a	886.6 ± 16.4a	568.3 ± 20.4c	454.4 ± 18.0d	399.0 ± 13.3b
	M2	616.2 ± 12.4ab	835.6 ± 15.3abc	607.9 ± 19.0abc	498.9 ± 18.7ab	427.4 ± 8.9ab
	M3	625.5 ± 15.1ab	868.1 ± 16.4ab	624.1 ± 14.7a	517.2 ± 12.4a	432.5 ± 9.1a
	M4	617.6 ± 11.8ab	853.2 ± 12.7ab	616.7 ± 14.4ab	506.7 ± 27.3ab	423.7 ± 22.3ab
	M5	605.1 ± 22.9ab	829.2 ± 11.1bc	597.7 ± 15.1abc	492.1 ± 15.5abc	414.5 ± a19.2b
	M6	594.9 ± 5.9b	798.8 ± 19.0c	572.2 ± 11.0bc	459.8 ± 26.8cd	400.5 ± 17.7b

In 2019−2020, the measurements at the jointing and booting stage were not performed due to the outbreak of COVID-19. Means within each column and year followed by different lowercase letters were significant at *p*< 0.05.

#### 3.2.2 Dynamics of dry matter accumulation

At the over-wintering and jointing stages, M1 achieved the highest DMA, which was significantly higher than M2. However, the accumulation rate of dry matter in M1 decreased from booting to maturity stage, resulting in the lowest DMA at the maturity stage in 2017−2021 (**Table 5**). DMA in M2 and M3 showed no advantage before booting stage, but it was significantly higher than that in CK and M1 at the maturity stage due to the rapid increase of DMA post-anthesis. Compared with CK, DMA at maturity and post-anthesis in M2 increased by an average of 4.02% and 19.95% in the two years, respectively, while that in M3 increased by 6.20% and 19.36%, respectively. With N reduction by 10%−30%, DMA in M4−M6 showed a gradual decreasing trend at different stages, but there was no significant difference in DMA between M3, M4 and M5. Compared with M3, M6 significantly decreased DMA post-anthesis by 12.16% and 15.27% in 2019−2020 and 2020−2021, respectively.

**Table 5:** Dynamics of dry matter accumulation (DMA) of wheat in different N treatments in 2017–2021.

Year	Treatment	DMA (kg ha^−1^)					DMA post-anthesis (kg ha^−1^)
		Over-wintering	Jointing	Booting	Anthesis	Maturity	
2017−2018	CK	647.3 ± 22.8a	3260.9 ± 112.8b	9618.7 ± 154.0a	13248.3 ± 149.8a	18548.4 ± 239.0b	5300.1 ± 388.8b
	M1	652.4 ± 11.9a	4071.0 ± 129.7a	9633.9 ± 185.8a	12274.7 ± 75.4c	17602.1 ± 165.7c	5327.3 ± 241.1b
	M2	542.6 ± 15.5b	3410.5 ± 64.8b	9502.0 ± 108.3b	12586.6 ± 61.2b	19284.4 ± 205.9a	6697.8 ± 367.2a
	M3	580.8 ± 10.9b	3452.9 ± 81.4b	9571.2 ± 4155.6ab	13026.0 ± 55.9a	19699.3 ± 196.3a	6673.3 ± 252.3a
2018−2019	CK	785.0 ± 32.9bc	3365.2 ± 10.4c	9113.8 ± 174.3ab	13212.7 ± 271.9b	19959.4 ± 374.8c	6746.7 ± 102.9b
	M1	915.6 ± 50.3a	4862.3 ± 147.1a	9176.1 ± 300.5ab	12272.6 ± 97.47c	18883.6 ± 292.4d	6611.0 ± 194.9b
	M2	749.0 ± 5.9c	4254.6 ± 30.1b	8747.9 ± 175.4b	13113.2 ± 160.57b	20772.2 ± 429.9b	7659.0 ± 269.4a
	M3	821.7 ± 25.34b	4399.1 ± 72.3b	9571.5 ± 242.2a	13583.8 ± 263.6a	21194.3 ± 378.5a	7610.5 ± 115.0a
2019−2020	CK	749.4 ± 22.1ab	−	−	13135.9 ± 116.2b	20013.7 ± 578.9bc	6877.8 ± 462.7bc
	M1	795.3 ± 33.7a	−	−	12603.9 ± 130.6c	19088.7 ± 61.2c	6484.9 ± 69.4c
	M2	699.7 ± 19.2bc	−	−	13528.7 ± 273.8ab	21341.9 ± 286.6a	7813.2 ± 120.8a
	M3	734.9 ± 15.3b	−	−	13860.1 ± 64.5a	21610.3 ± 545.1a	7750.2 ± 480.7a
	M4	726.0 ± 22.6bc	−	−	13833.7 ± 133.9a	21472.4 ± 439.4a	7638.8 ± 305.5a
	M5	705.3 ± 8.4bc	−	−	13347.7 ± 125.7b	20772.8 ± 636.8ab	7425.1 ± 511.1ab
	M6	673.6 ± 14.7c	−	−	12381.0 ± 230.8c	19188.7 ± 361.8c	6807.7 ± 131.0bc
2020−2021	CK	634.8 ± 38.1ab	3115.0 ± 98.7b	9531.8 ± 166.3ab	13148.4 ± 22.7ab	18628.9 ± 334.3bc	5480.53 ± 357.0bc
	M1	681.2 ± 17.5a	3607.4 ± 160.0a	9342.68 ± 86.8abc	12329.2 ± 310.4c	17585.8 ± 233.8d	5256.6 ± 86.6c
	M2	592.3 ± 24.6b	3231.0 ± 230.1b	9005.8 ± 158.7cd	13196.2 ± 257.0a	19612.3 ± 336.5a	6416.1 ± 79.5a
	M3	620.1 ± 6.9ab	3350.2 ± 49.9ab	9705.7 ± 286.7a	13349.1 ± 187.4a	19625.4 ± 440.4a	6276.3 ± 253.1a
	M4	612.8 ± 39.9ab	3292.5 ± 166.3ab	9392.1 ± 30.3abc	13193.7 ± 90.7a	19373.1 ± 189.7ab	6179.5 ± 280.4a
	M5	602.7 ± 12.8b	3163.9 ± 44.1b	9179.9 ± 168.3bc	12939.2 ± 143.9ab	18902.9 ± 363.5ab	5963.7 ± 219.6ab
	M6	580.2 ± 23.6b	3014.6 ± 140.3b	8706.8 ± 177.6d	12624.5 ± 197.5bc	17942.1 ± 135.9cd	5317.6 ± 61.5c

In 2019−2020, the measurements at the jointing and booting stage were not performed due to the outbreak of COVID-19. Means within each column and year followed by different lowercase letters were significant at *p*< 0.05.

#### 3.2.3 Dynamics of leaf area index

Similar to the trend of stem and tiller number, LAI in M1 was the highest at the over-wintering and jointing stages, but decreased rapidly after booting stage, which was significantly lower than that in M2 and M3 at the anthesis and milk-ripening stages (**Table 6**). LAI in M2 and M3 were significantly higher than that in CK at the milk-ripening stage, with a four-year average increase of 34.44% and 28.76%, respectively. In 2019−2021, M4 showed no significant difference in LAI at anthesis and milk-ripening stages compared with M3. However, M5 and M6 significantly decreased LAI at both anthesis and milk-ripening stages compared with M3, which decreased by 8.81% and 17.60% at the milk-ripening stage, respectively.

**Table 6:** Dynamics of leaf area index (LAI) of wheat in different N treatments in 2017–2021.

Year	Treatment	Over-wintering	Jointing	Booting	Anthesis	Milk-ripening
2017−2018	CK	0.83 ± 0.01ab	5.11 ± 0.04c	7.05 ± 0.03b	5.22 ± 0.13c	3.00 ± 0.01c
	M1	0.87 ± 0.03a	5.98 ± 0.02a	7.11 ± 0.01b	5.86 ± 0.03b	3.15 ± 0.01b
	M2	0.67 ± 0.01c	5.53 ± 0.13b	7.11 ± 0.01b	6.57 ± 0.04a	4.40 ± 0.01a
	M3	0.76 ± 0.04b	5.58 ± 0.04b	7.34 ± 0.07a	6.81 ± 0.10a	4.37 ± 0.06a
2018−2019	CK	1.13 ± 0.11ab	3.97 ± 0.17b	6.31 ± 0.18b	5.03 ± 0.08c	3.06 ± 0.08b
	M1	1.31 ± 0.03a	4.67 ± 0.06a	6.29 ± 0.07b	5.04 ± 0.12c	3.18 ± 0.15b
	M2	1.04 ± 0.03b	4.22 ± 0.16ab	6.43 ± 0.06ab	5.56 ± 0.10b	4.05 ± 0.21a
	M3	1.17 ± 0.10ab	4.46 ± 0.22ab	6.75 ± 0.06a	5.91 ± 0.08a	3.75 ± 0.07a
2019−2020	CK	1.23 ± 0.04d	−	−	5.41 ± 0.06c	3.31 ± 0.07d
	M1	1.33 ± 0.06a	−	−	5.18 ± 0.06d	3.22 ± 0.11d
	M2	1.07 ± 0.04cd	−	−	5.86 ± 0.04a	4.32 ± 0.08a
	M3	1.13 ± 0.05c	−	−	5.96 ± 0.05a	4.15 ± 0.08ab
	M4	1.08 ± 0.02cd	−	−	5.89 ± 0.03a	3.94 ± 0.07b
	M5	1.01 ± 0.04d	−	−	5.61 ± 0.08b	3.65 ± 0.04c
	M6	0.93 ± 0.03e	−	−	5.28 ± 0.11cd	3.30 ± 0.06d
2019−2020	CK	1.12 ± 0.03b	3.96 ± 0.04e	6.58 ± 0.02abc	5.07 ± 0.07c	3.08 ± 0.04d
	M1	1.33 ± 0.05a	4.67 ± 0.09a	6.37 ± 0.07c	4.97 ± 0.10c	3.06 ± 0.04d
	M2	0.99 ± 0.04c	4.24 ± 0.06cd	6.64 ± 0.05ab	5.67 ± 0.08ab	3.94 ± 0.05a
	M3	1.18 ± 0.05b	4.46 ± 0.09b	6.79 ± 0.12a	5.84 ± 0.04a	3.74 ± 0.07b
	M4	1.10 ± 0.05b	4.31 ± 0.08bc	6.63 ± 0.08ab	5.68 ± 0.10ab	3.66 ± 0.08bc
	M5	0.98 ± 0.04c	4.16 ± 0.05cd	6.42 ± 0.11bc	5.49 ± 0.08b	3.53 ± 0.05c
	M6	0.91 ± 0.03c	4.09 ± 0.04de	6.12 ± 0.08d	5.09 ± 0.11c	3.18 ± 0.04d

In 2019−2020, the measurements at the jointing and booting stage were not performed due to the outbreak of COVID-19. Means within each column and year followed by different lowercase letters were significant at *p*< 0.05.

#### 3.2.4 Total grain number

**Figure 2:**
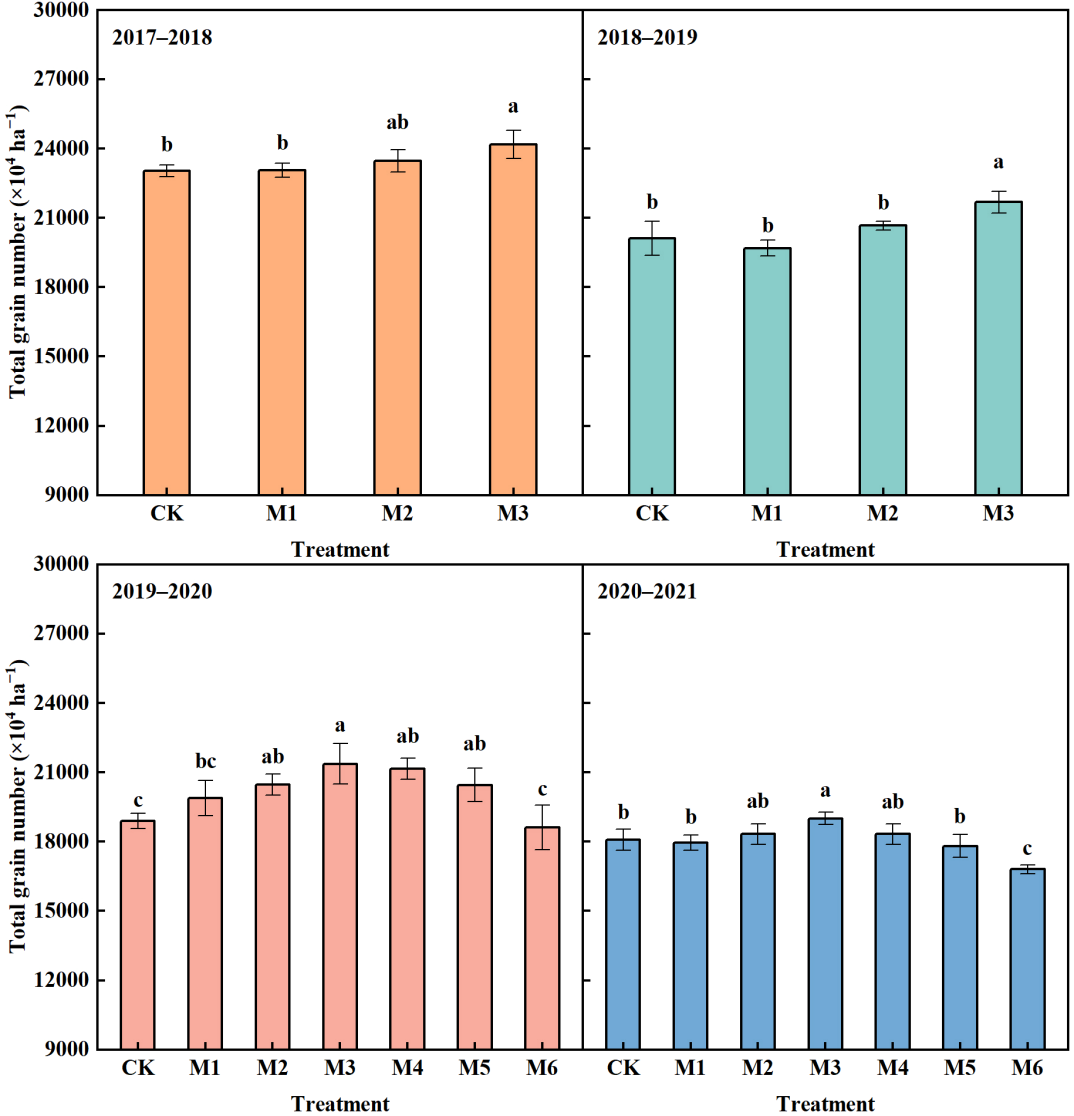
Total grain number of wheat in different N treatments in 2017–2021. The different lowercase above bars indicates significant difference (*P*< 0.05) among treatments.

Among different treatments, M3 achieved the highest total grain number, which increased by 4.93%, 7.82%, 13.12% and 4.93% compared with CK in 2017−2021, respectively (**Figure 2**). The total grain number in M2 showed a higher trend than CK, but the significant difference was observed only in 2019−2020. With N reduction by 10%−30%, the total grain number in M4−M6 showed a gradual decreasing trend, but only M6 was observed to significantly decrease the total grain number compared with M3.

### 3.3 Response of nitrogen uptake and nitrogen efficiency to different nitrogen treatments

#### 3.3.1 Nitrogen uptake at different growth stages

**Figure 3:**
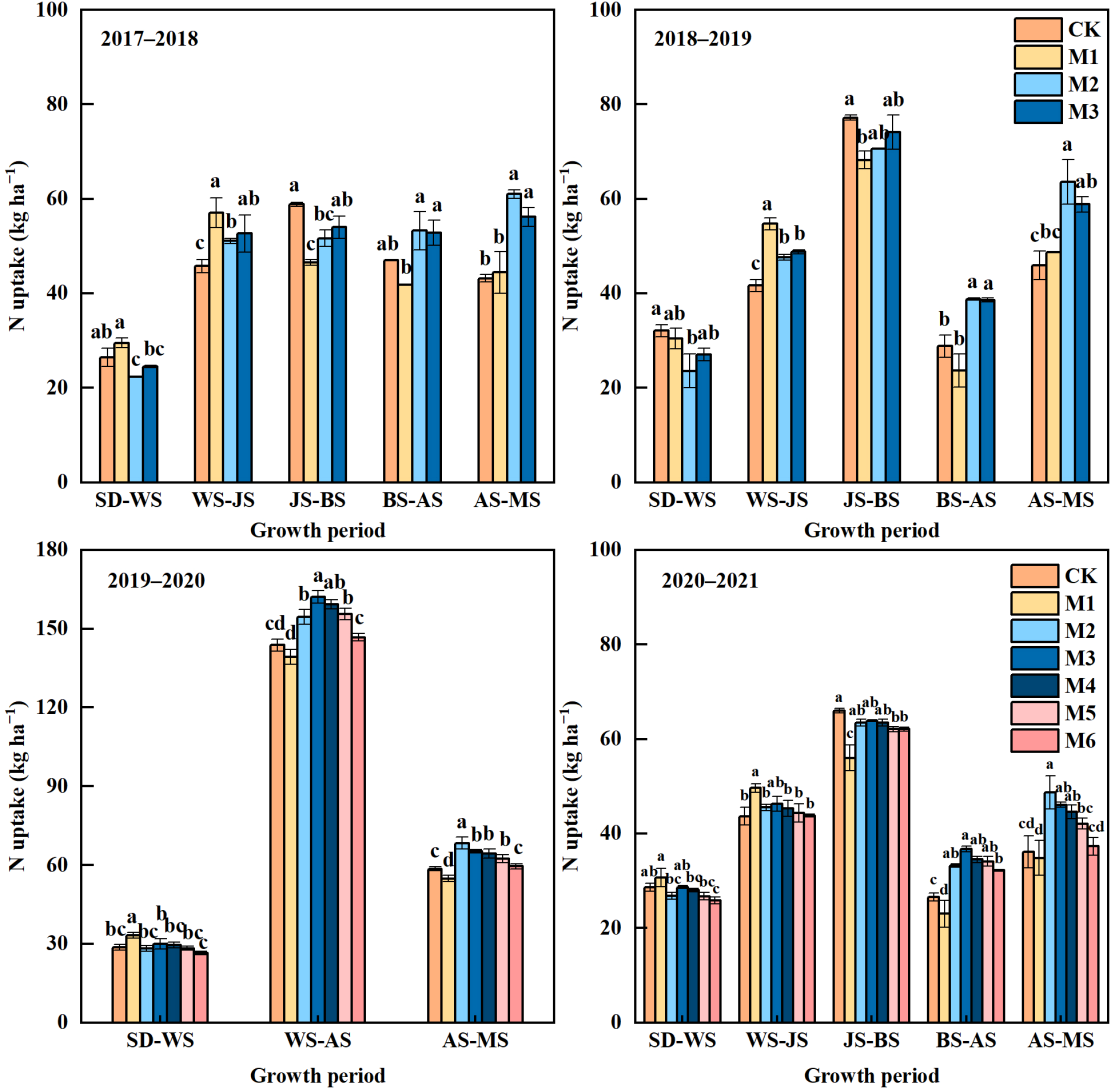
Nitrogen uptake of wheat at different growth periods in different N treatments in 2017–2021. SD, Sowing date; WS, Over-wintering stage; JS, Jointing stage; BS, Booting stage; AS, Anthesis stage; MS, Maturity stage. In 2019−2020, the measurements at the jointing and booting stage were not performed due to the outbreak of COVID-19. The different lowercase above bars indicates significant difference (*P*< 0.05) among treatments.

The N uptake by wheat was significantly affected by different N treatments (**Figure 3**). M1 showed a trend of improving N uptake compared with M2 and M3 from seeding to jointing stage. However, due to insufficient N supply after jointing, the N uptake in M1 was significantly lower than that in M2 and M3 from booting to maturity stage. Compared with CK, M2 and M3 decreased N uptake before over-wintering, but significantly increased N uptake from booting to maturity stage.

With N reduction by 10%−30%, the N uptake in M4−M6 showed a gradual decrease trend at different growing periods (**Figure 3**). From anthesis to maturity stage, M3 showed no significant difference compared with M4 and M5, but was significantly higher than M6. In addition, the N uptake in M4 and M5 tended to be higher than that in CK from anthesis to maturity stage in 2019−2020 and from booting to maturity stage in 2020−2021.

#### 3.3.2 Nitrogen accumulation at maturity stage

**Figure 4:**
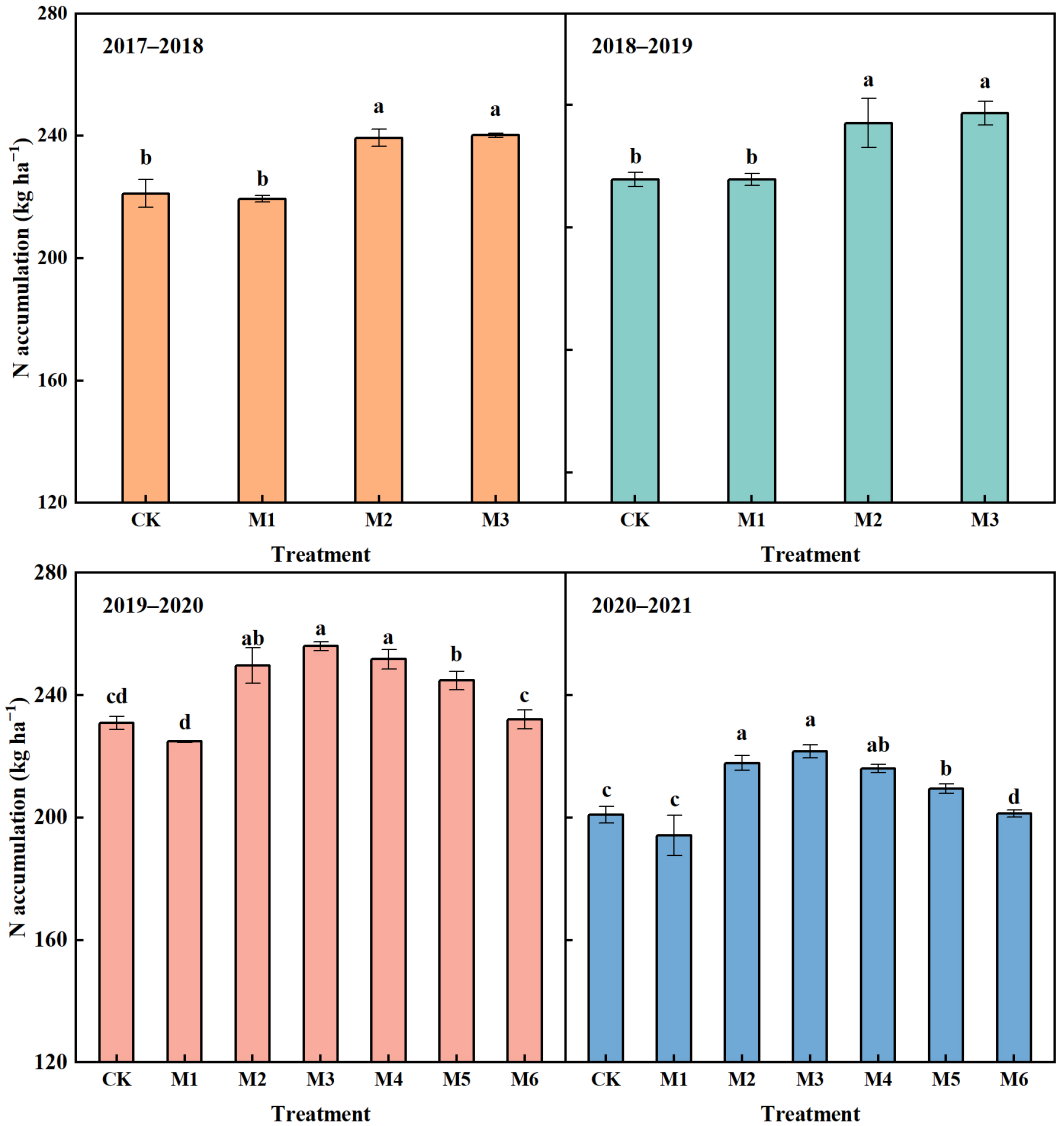
Nitrogen accumulation of wheat at maturity stage in different N treatments in 2017–2021. The different lowercase above bars indicates significant difference (*P*< 0.05) among treatments.

With the equal N application rate, the N accumulation in M1 showed no significant difference compared with CK in 2017−2021 (**Figure 4**). The N accumulation in M2 and M3 was significantly higher than that in CK in the four years, with an average increase of 8.23% and 9.83%, respectively. Compared with that in M1, the N accumulation in M2 and M3 increased by 10.13% and 11.76% on average in 2017−2021, respectively.

The N accumulation in M4−M6 showed a continuous downward trend with the decrease of N application rate (**Figure 4**). In 2019−2021, the average N accumulation in M4, M5 and M6 decreased by 2.12%, 4.96% and 9.28%, respectively, compared with that in M3. While N was reduced by 20%, N accumulation in M5 was significantly lower than that in M3, but was significant higher than that in CK. With N reduced by 30%, M6 significantly decreased N accumulation compared with CK in 2020−2021, but showed no significant difference compared with CK in 2019−2020.

#### 3.3.3 Nitrogen recovery efficiency

**Figure 5:**
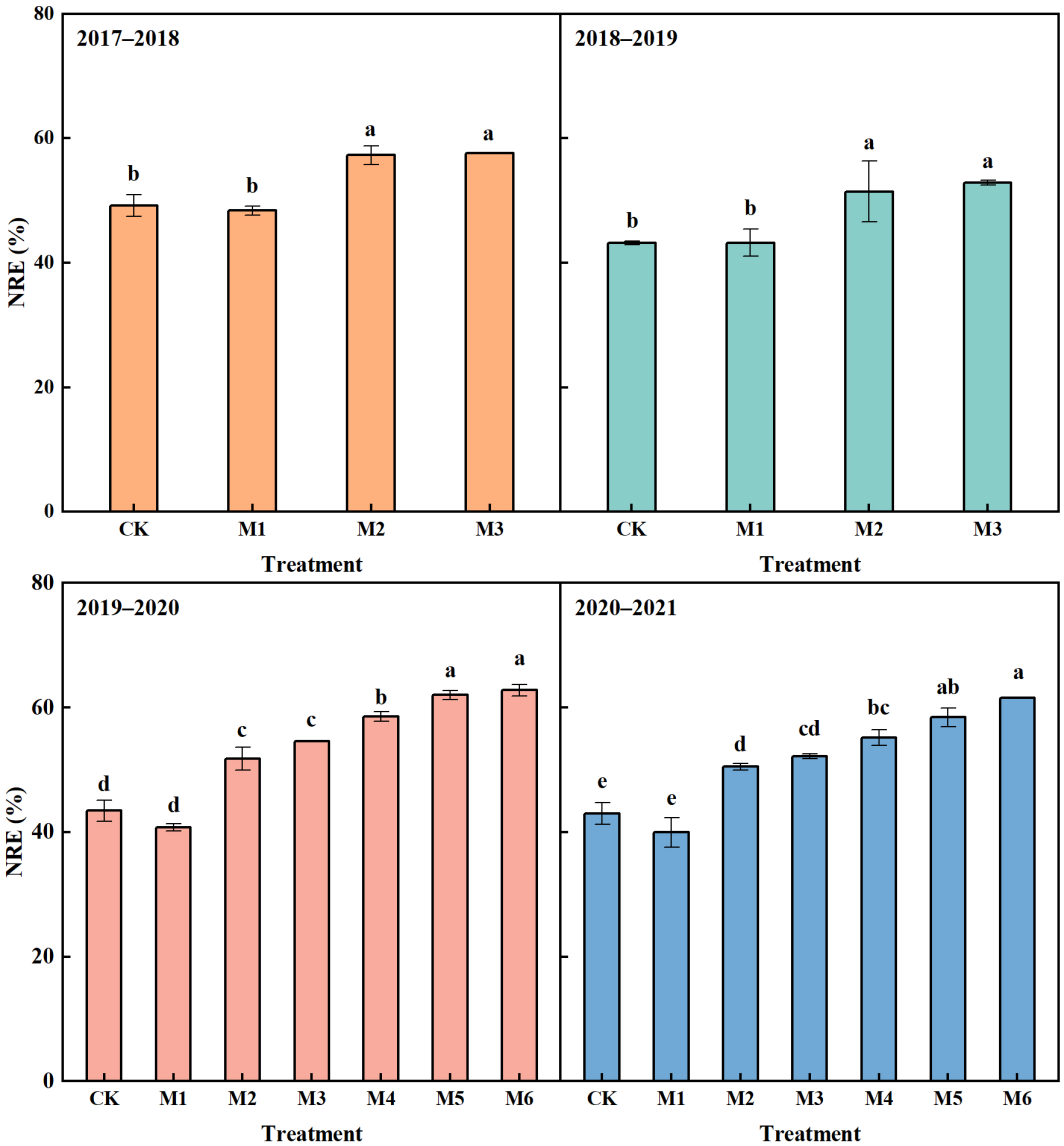
Nitrogen recovery efficiency (NRE) in different N treatments in 2017–2021. The different lowercase above bars indicates significant difference (*P<* 0.05) among treatments.

With the equal N application rate, M1 achieved the lowest NRE, which decreased by 1.63% on average compared with CK in 2017−2021 (**Figure 5**). NRE in M3 was the highest, which was significantly higher than that in CK in 2017−2021, with an increase of 8.44%, 9.62%, 11.17% and 9.16 in the four years, respectively. NRE in M2 had no significant difference compared with M3, which was also significantly higher than that in CK, with an average increase of 8.03% in the four years.

The NRE showed a continuous increase trend with the reduction of N application rate (**Figure 5**). Compared with that in M3, the NRE in M4 increased by 3.47% on average in 2019−2021, but the significant difference was observed only in 2019−2020. M5 and M6 significantly improved NRE compared with M3 in 2019−2021, with an average increase of 6.82% and 8.79%, respectively. Moreover, the NRE in M4, M5 and M6 significantly increased by 13.63%, 16.98% and 18.95% compared with CK, respectively.

#### 3.3.4 Nitrogen agronomic efficiency

**Figure 6:**
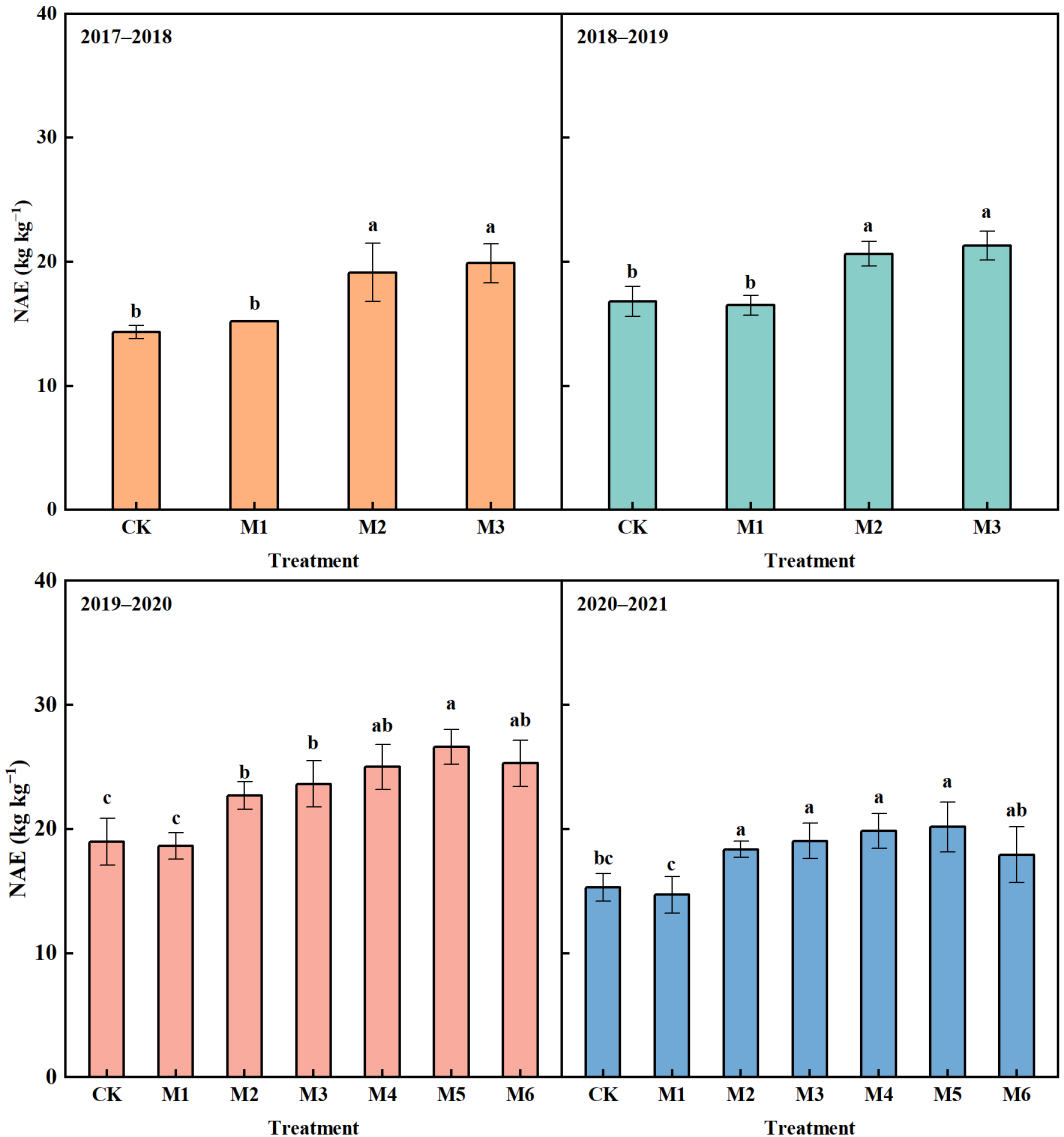
Nitrogen agronomic efficiency (NAE) in different N treatments in 2017–2021. The different lowercase above bars indicates significant difference (*P*< 0.05) among treatments.

With the equal N application rate, the NAE in M1 showed no significantly difference compared with that in CK in 2017−2021 (**Figure 6**). M2 and M3 significantly increased NAE compared with CK in 2017−2021, with an average increase of 24.04% and 38.6% in the four years, respectively.

With N reduction by 10%−20%, the NAE in M4−M5 showed an upward trend compared with M3 (**Figure 6**). M5 achieved the highest NAE, which increased by 12.67% and 5.90% compared with M3 in 2019−2020 and 2020−2021, respectively. M4, M5 and M6 significantly increased NAE compared with CK in the two years, with an average increase of 30.79%, 36.12% and 25.32%, respectively.

### 3.4 Response of wheat economic benefits to different nitrogen treatments

With the equal N application rate, the differences in economic benefits under different patterns were mainly caused by output, N fertilizer cost and topdressing labor cost (**Table 7**). In 2017−2019, M1 saved topdressing labor input, but increased N fertilizer cost compared with CK, resulting in a total cost increase of 279.48 CNY ha^−1^. Compared with CK, M2 increased the total cost by 429.48 CNY ha^−1^, which saved twice topdressing labor input but had the highest N fertilizer cost. Due to the partial substitution of PCU with urea, M3 decreased the total cost compared with M2, which only increased by 64.74 CNY ha^−1^ compared with CK. In 2019−2021, the cost in different patterns increased due to the increase in N fertilizer price, but the trend was consistent with that in 2017−2019. Except for 2017−2018, the net benefit in M1 showed a downward trend compared with CK in the four years, with an average decrease of 357.71 CNY ha^−1^. Although the cost of fertilization increased to varying degrees, the average net benefit in M2 and M3 in 2017−2021 increased by 1566.02, and 2342.18 CNY ha^−1^, respectively, compared with CK.

**Table 7:** Economic benefit of wheat in different N treatments in 2017–2021.

Year	Treatment	Output (CNY ha^−1^)	N fertilizer cost (CNY ha^−1^)	Topdressing labor cost (CNY ha^−1^)	Net benefit (CNY ha^−1^)	Cost increase (CNY ha^−1^)	Benefit increase (CNY ha^−1^)
2017− 2018	CK	16719.20	1020.52	450.00	9322.69	−	−
	M1	17168.58	1750.00	0.00	9492.58	279.48	169.89
	M2	19245.50	1750.00	150.00	11419.50	429.48	2096.82
	M3	19635.25	1385.26	150.00	12173.99	64.74	2851.30
2018− 2019	CK	19570.66	1020.52	450.00	12174.14	−	−
	M1	19413.13	1750.00	0.00	11737.13	279.48	−437.02
	M2	21531.52	1750.00	150.00	13705.52	429.48	1531.38
	M3	21865.89	1385.26	150.00	14404.63	64.74	2230.49
2019− 2020	CK	21622.20	1312.10	450.00	13934.10	−	−
	M1	21438.00	2100.00	0.00	13412.00	337.90	−522.10
	M2	23646.30	2100.00	150.00	15470.30	487.90	1536.20
	M3	24163.06	1706.05	150.00	16381.01	93.95	2446.91
	M4	23545.69	1535.44	150.00	15934.25	−76.65	2000.15
	M5	22891.75	1364.84	150.00	15450.91	−247.26	1516.81
	M6	20930.79	1194.23	150.00	13660.56	−417.86	−273.54
2020− 2021	CK	17981.66	1312.10	450.00	10293.57	−	−
	M1	17677.93	2100.00	0.00	9651.93	337.90	−641.63
	M2	19569.23	2100.00	150.00	11393.23	487.90	1099.66
	M3	19915.64	1706.05	150.00	12133.59	93.95	1840.02
	M4	19307.63	1535.44	150.00	11696.18	−76.65	1402.61
	M5	18416.35	1364.84	150.00	10975.52	−247.26	681.95
	M6	16573.33	1194.23	150.00	9303.09	−417.86	−990.48

Due to saving N input, M4, M5 and M6 decreased the total cost compared with CK. M4 and M5 decreased the total output compared with M3, but increased net benefit compared with CK, with an average increase of 1701.38 and 1099.38 CNY ha^−1^ in 2019−2021, respectively. Although it had the lowest total cost, M6 sharply reduced the output, contributing to an average decrease of 632.01 CNY ha^−1^ in net benefit compared with CK in the two years.

## 4 Discussion

The key to increasing wheat yield and NUE lies in building suitable populations to coordinate the competition among individual plants. The suitable agronomic characters such as appropriate spike number, higher DMA post-anthesis and appropriate LAI are important prerequisites for achieving high yield (Tao et al., 2018; Li et al., 2021b). N fertilizer is the most direct cultivation measure to regulate population structure and promote yield improvement. It has been confirmed that reasonable N supply during the growth period of wheat was beneficial for cultivating strong seedlings, promoting tiller and panicle formation, improving DMA post-anthesis, and increasing the grain weight (Duan et al., 2019; Zhang et al., 2020; Ding et al., 2023). In this study, M1 had the highest stem and tiller number, DMA and LAI at the over-wintering and jointing stages. However, it was observed that a large number of tillers died after jointing, and LAI decreased rapidly after booting in M1 (**Tables 4**−**6**). This was consistent with the findings of Li et al. (2021b), who believed that CRNF applied once pre-sowing could easily lead to excessive nutrient supply in the early stage but insufficient N supply in the middle and later stages, resulting in rapid growth during the vegetative growth period but poor development during the reproductive growth period, thereby limiting DMA post-anthesis. It has been confirmed that the accumulation of photosynthetic products post-anthesis largely determined the grain yield of wheat (Liu et al., 2020). The photosynthesis of leaves is a key driving force for the accumulation and distribution of assimilates in plants, while the sustained function of leaves during the filling period is the basis for promoting photosynthetic performance and assimilate accumulation, which largely determines grain weight and yield (Makino, 2011; Gaju et al., 2016). The low spike number and LAI post-anthesis in M1 were obviously detrimental to the production and accumulation of photosynthetic products, which restricted the yield potential.

Previous studies have also pointed out that the increase in wheat yield in high-yield fields is mainly limited by assimilating sink, and increasing the grain number per unit area is an important strategy to increase yield potential (Reynolds et al., 2012; Alonso et al., 2018). In source-sink transition, the total grain number reflects the capacity of sink, and drives the accumulation of photosynthetic products post-anthesis (Makino, 2011; Sierra-Gonzalez et al., 2021). Although M2 and M3 showed no advantage in DMA and LAI before jointing stage compared with M1 and CK, the higher total grain number and DMA post-anthesis provided a possibility for achieving high yield (**Figure 2**). Compared with M1, the reasonable N supply in M2 and M3 increased the spike number and total grain number, and maintained higher LAI post-anthesis, contributing greatly to the DMA post-anthesis, which was the key to improving grain yield.

N absorption and utilization is an important basis for crop growth, which is closely related to yield formation (Yang et al., 2016). The application of CRNF has been proven to reduce the risk of soil N loss t and promote the N uptake by wheat, thus improving NUE (Zheng et al., 2016b; Lü et al., 2023; Zhang et al., 2024). Some studies believe that one-time application of CRNF could simultaneously meet the N demand in the early and later growth stages of wheat, thereby promoting the N absorption and utilization of plants and improving the N harvest index (Zheng et al., 2016a; Shen et al., 2022; Wang et al., 2023). However, our observation showed that M1 promoted the wheat N uptake before jointing, but significantly decreased N uptake from jointing to maturity stage compared with M2 and M3, contributing to significantly lower N accumulation at the maturity stage (**Figures 3**, **4**). Li et al. (2021b) showed that the nutrient release of CRNF applied once failed to match the N demand of wheat, which could impede wheat N uptake in the later growth stage and decrease NUE. In the process of wheat growth, appropriate N supply according to the law of wheat N demand is an important guarantee for plant growth and development (Lu et al., 2015). In fact, the N uptake by wheat is relatively low due to the small size at seedling stage, but is more vigorous after jointing. Excessive N supply in the early stage was difficult to supply the plant growth in the later stage, but instead promoted the nitrate enrichment in soil, exacerbating the risk of N loss (Zheng et al., 2016b; Shakoor et al., 2018), which could explain why one-time application of CRNF pre-sowing failed to achieve the expected yield.

Geng et al. (2015) and Yang et al. (2012) pointed out that the key to improving NUE for CRNF was that it could supply N sustainably and steadily to reduce N loss at the seedling stage, and meet the nutrient demand of crops throughout the growth period. Our previous study has also confirmed that twice-split application of CRNF was conducive to controlling the inorganic N content in soil at the seedling stage, and maintaining a high level of inorganic N from jointing to maturity stage, thus improving the soil N supply capacity at the middle and later growth stage of wheat (Ma et al., 2021). This finding could explain the higher N uptake in M2 compared with M1 from jointing to maturity stage (**Figure 3**). With the growth of crops, CRNF continuously release N. Crop roots could directly absorb sufficient soil N in the later stage, which was also conducive to N accumulation and distribution to grain, thus promoting grain filling and yield formation (Li et al., 2021b; Gao et al., 2022). Compared with M2, M3 slightly decreased N uptake from booting to maturity stage, but the difference was not significant, which indicated that compared with all PCU applied twice, partial replacement of PCU with common urea could also meet the N demand of wheat, and contribute to N accumulation of wheat (**Figures 3**, **4**). A study on rice by Tang et al. (2007) found that the application of CRNF promoted the vitality of roots and thus improved the N uptake by plant. It could be inferred that sufficient N supply in M2 and M3 could also delay the senescence of wheat roots in the later stage of growth, and promote the soil N absorption and utilization by roots, thereby leading to higher N uptake by plants from booting to maturity stage. Therefore, our future research will focus on the regulation effect of CRNF application on the morphology and physiology of wheat root. Due to the remobilization of N, the insufficient N supply post-anthesis could induce the senescence of leaves, leading to the deficiency of assimilated production and thus restricting the grain filling process (Borrell et al., 2001; Kitonyo et al., 2018). In this study, compared with that in M1, the higher LAI in M2 and M3 at the milk-ripening stage could be closely related to higher N uptake and root activity post-anthesis, which contributed to significantly higher DMA post-anthesis and 1000-grain weight.

There is no doubt that urea has played an important role in improving wheat yield. However, with the continuous increase of urea consumption, the problems of low NUE and low benefit return have always been difficult to overcome (Srivastava et al., 2018). Our results showed that, with the equal N application rate, M2 and M3 significantly improved NRE and NAE compared with CK, which was mainly attributed to the increase of N absorption by plant from booting to maturity stage (**Figures 3**−**6**). Li et al. (2021b) also reported similar results, which thought that although the nitrogen supply level of composite fertilizers was lower than that of one-time basal application of CRNF at the seedling stage, it also alleviated a large amount of nitrogen loss at this stage, thus ensuring sufficient nitrogen absorption of plants after booting and achieving higher nitrogen efficiency during the whole growth period of wheat.

Zheng et al. (2016b) reported that compared with twice-split application of urea, CRNF applied once pre-sowing increased the grain yield by 3.2%−10.1% and NUE by 36.2%−45.4%. However, our observation found that M1 failed to increase the yield and NRE compared with CK, which could be due to the differences in urea application methods and planting systems (**Table 3**). Urea applied in four times cater to fertilization habits of farmers during wheat cultivation process in the local region. Compared with CK, M2 and M3 increased the yield by 8.83%−17.44%. M3 achieved the highest grain yield in the four years, but showed no significant difference from M2 (**Table 3**). It could be found that the yield increase in M2 and M3 was mainly due to the co-increase in spike number and 1000-grain weight. The sufficient N supply in M2 and M3 at the later growth stage not only reduced the death of tillers, but also promoted the assimilation accumulation and grain filling (Ma et al., 2021). Shen et al. (2022) also confirmed that CRNF applied once pre-sowing caused insufficient N supply after jointing, which was detrimental to effective tiller-earing and limited the yield increase.

CRNF has shown great prospects in field crop production due to the characteristics of labor-saving and efficiency-enhancing (Zheng et al., 2016a). Unfortunately, the high cost has always been the main factor limiting the promotion and application of CRNF (Davidson and Gu, 2012). Therefore, the willingness of producers to adopt CRNF depends on whether the yield returns and labor savings can compensate for the increased fertilizer cost. In this study, M1 avoided the input of artificial topdressing and similar yield compared with CK, but increased the fertilizer cost, resulting in an average net benefit decrease of 357.71 CNY ha^−1^ (**Table 7**). As a result, CRNF applied once pre-sowing was not suitable for large-scale production but recommended for use in areas with labor shortages. To control the fertilizer cost, Zheng et al. (2017) proposed a fertilization strategy of mixing controlled-release urea with 30% ordinary urea, which could increase the net profit by 15.4%−21.8% compared with ordinary urea treatment with the equal N rate. Zhang et al. (2023) showed that a mixture of CRNF with urea could increase wheat yield by 3.12%−14.62% under different N application rates. Farmaha and Sims (2013b) also believed that the application of the mixed fertilizer with CRNF and urea was beneficial to improve the economic returns of producers. Our study confirmed that the net benefit in M2 and M3 was significantly improved compared with that in CK, with an average increase of 1566.15 and 2342.18 CNY ha^−1^ in the four years, respectively (**Table 7**), which was attributed to the increase in output and the saving of twice topdressing inputs. Compared with M2, M3 showed no significant difference in grain yield, but achieved obvious higher net benefit, which could be explained by the lower fertilizer cost by blending PCU with urea. Our observation also found that, with N reduction by 10%−20%, M4 and M5 significantly increased NRE and NAE compared with CK without reducing yield; while N reduced by 30%, M6 significantly increased NRE but decreased yield compared with CK, contributing to reducing net benefit. The results showed that compared with urea multiple application, twice-split application of PCU combined with urea could synergistically improve wheat yield, NRE and net benefit, and significantly increased NRE without sacrificing yield and benefit compared with traditional fertilization strategy within the range of 20% N reduction. In areas with high N fertilizer consumption and high soil fertility, the above fertilization strategy is strongly recommended, which can save many production costs and labor input, and alleviate the environmental pollution caused by N loss in farmland.

## 5 Conclusion

Compared with multiple application of urea, twice-split application of 100% polymer-coated urea or 50% polymer-coated urea combined with 50% common urea was conducive to building the appropriate population structure, delaying the decline of leaf area index after booting, increasing dry matter accumulation post-anthesis, and promoting the synergistic increase in spike number and 1000-grain weight, thereby significantly increasing grain yield and nitrogen use efficiency while controlling the fertilization frequency. Compared with 100% polymer-coated urea, twice-split application of polymer-coated urea combined with common urea achieved higher net benefit due to the reduction of fertilizer cost, and showed an obvious potential to improve nitrogen use efficiency while slightly increasing yield and profit compared with urea multiple application under the condition of 20% nitrogen reduction, which was suitable for large-scale application on wheat grown in paddy soil.

## Data availability statement

The original contributions presented in the study are included in the article/supplementary material. Further inquiries can be directed to the corresponding author.

## Author contributions

QM: Investigation, Visualization, Writing – original draft. RT: Investigation, Writing – review & editing. WJ: Investigation, Writing – review & editing. MZ: Conceptualization, Writing – review & editing. CL: Conceptualization, Writing – review & editing. JD: Conceptualization, Writing – review & editing. WG: Conceptualization, Supervision, Writing – review & editing. GZ: Conceptualization, Supervision, Writing – review & editing. XZ: Funding acquisition, Supervision, Writing – review & editing.
